# Supplementary material for: Arsenic Trioxide and (−)-Gossypol Synergistically Target Glioma Stem-Like Cells via Inhibition of Hedgehog and Notch Signaling
Source: Cancers (Basel). 2019 Mar 12;11(3):350. doi: 10.3390/cancers11030350 (PMC6468469; doi:10.3390/cancers11030350)

Supplementary Materials: Arsenic Trioxide and (−)-Gossypol Synergistically Target Glioma Stem-Like Cells Via Inhibition of Hedgehog and Notch Signaling

**Benedikt Linder, Andrej Wehle, Stephanie Hehlgans, Florian Bonn, Ivan Dikic, Franz Rödel, Volker Seifert and Donat Kögel**


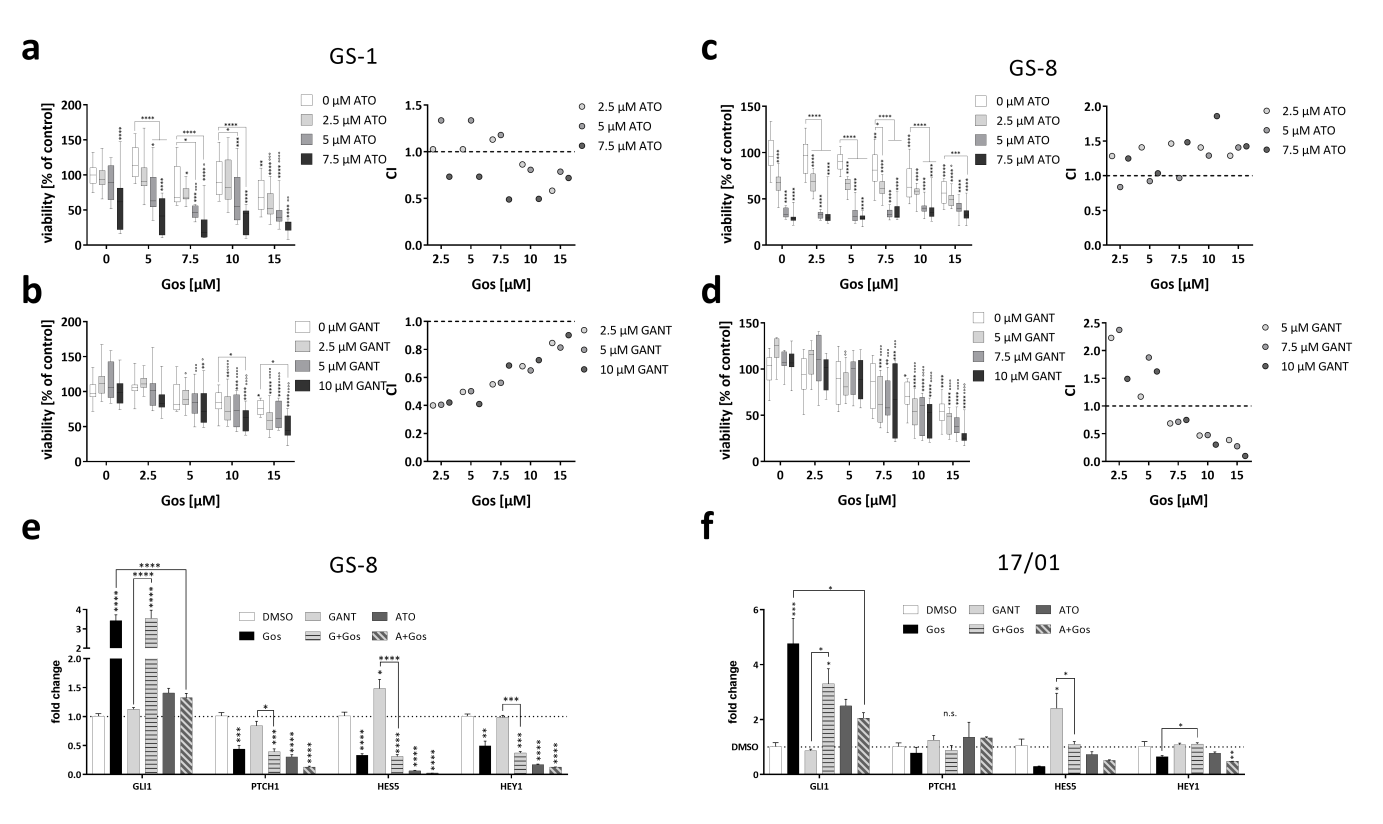


**Figure S1.** Synergistic inhibition of viability of glioma stem-like cells with a restricted and full stem-like phenotype via inhibition of Hh and Notch signaling. Viability (MTT) assay of (**a** and **b**) GS-1 restricted or (**c** and **d**) GS-8 full glioma stem like cells after treatment for 24 h with increasing concentrations (**a** and **c**) ATO or (**b** and **d**) GANT in combination with Gos. (**a** and **b**), right side) The CI was calculated from the data obtained according to Chou et al. (21) using the non-constant ratio setting (CI > 1: antagonism; CI = 1: additive; CI < 1: synergism). (**e** and **f**) Bar graph of Taqman-based gene expression analysis of (**e**) GS-8 and (**f**) 17/02 after treatment with 5 µM Gos, 2.5 µM GANT or ATO or the combination of GANT or ATO with Gos (G + Gos; A + Gos). The MTT data are presented as Box-Plots (Tukey) of at least two experiments performed in 6 biological replicates. The CI value is given as a single value calculated from the summary of all experiments. Gene expression experiments were conducted in triplicates and performed three times (GS-8) and once (17/01) * *p* < 0.05; ** *p* < 0.01; *** *p* < 0.001; **** *p* < 0.0001 against solvent or as indicated. ° *p* < 0.05; °° *p* < 0.01; °°° *p* < 0.001; °°°° *p* < 0.0001 against GANT or ATO single treatment.


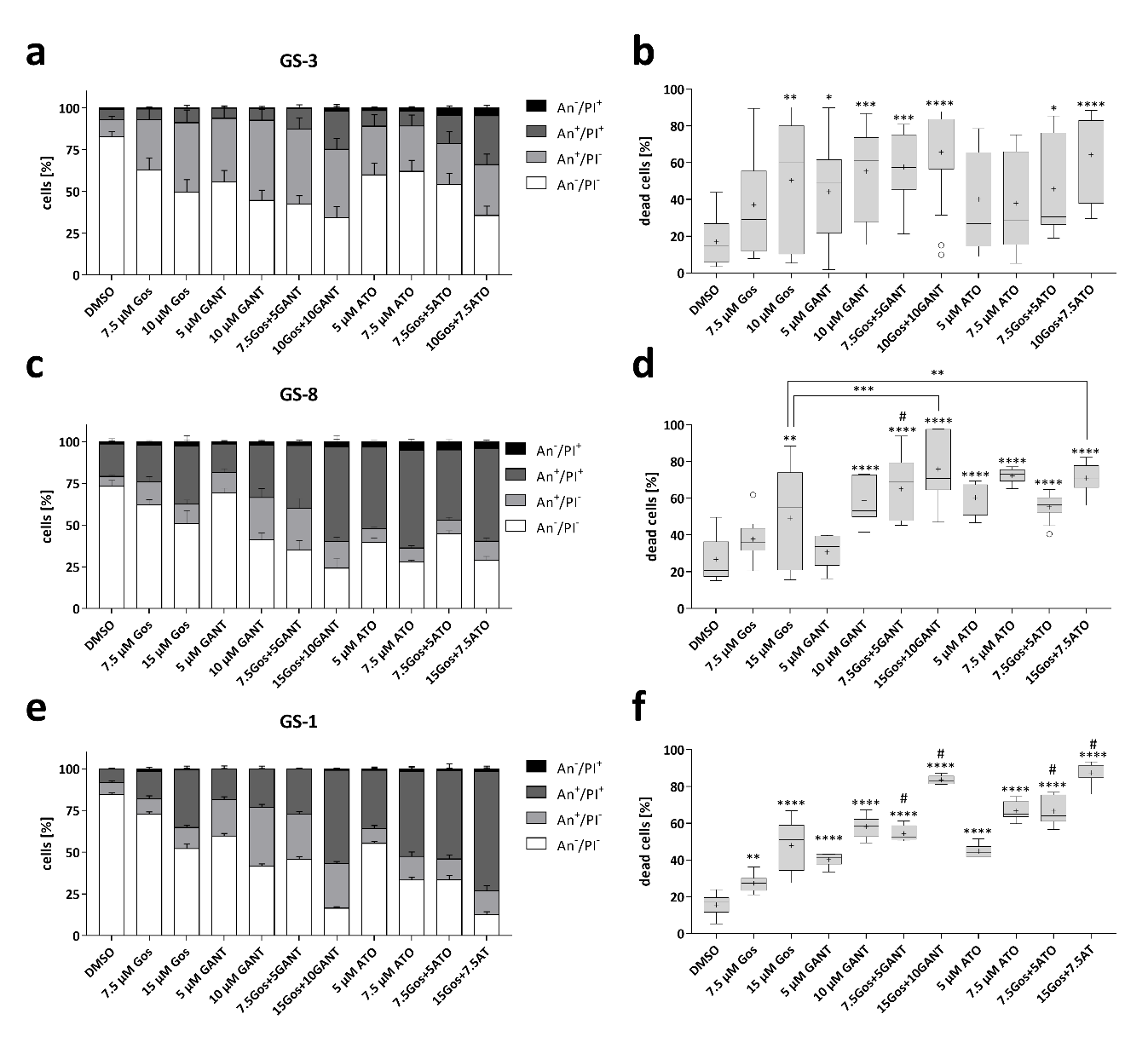


**Figure S2.** Synergistic induction of cell death after combined treatment with GANT or ATO with Gos. (**a**, **c** and **e**) stacked bar chart of (**a**) GS-3, (**b**) GS-8 and (**c**) the adherently growing GS line GS-1 after treatment for 24h with the drugs and concentrations as indicated. Box- Plots of the percentages of dead cells (100%—An-/PI-) of (**b**) GS-3, (**d**) GS-8 and (**f**) GS-1 after treatment as. Error bars in (**a** and **b**) are SEM. The graphs are the summary of at least three experiments performed in triplicates. The line in the Box-Plots represents the median, the plus-Symbol the mean * *p* < 0.05; ** *p* < 0.01; *** *p* < 0.001; **** *p* < 0.0001 against solvent; # *p* < 0.05 against both single treatments. One-way ANOVA followed by Tukey Post-Hoc-Test (GraphPad Prism 7).


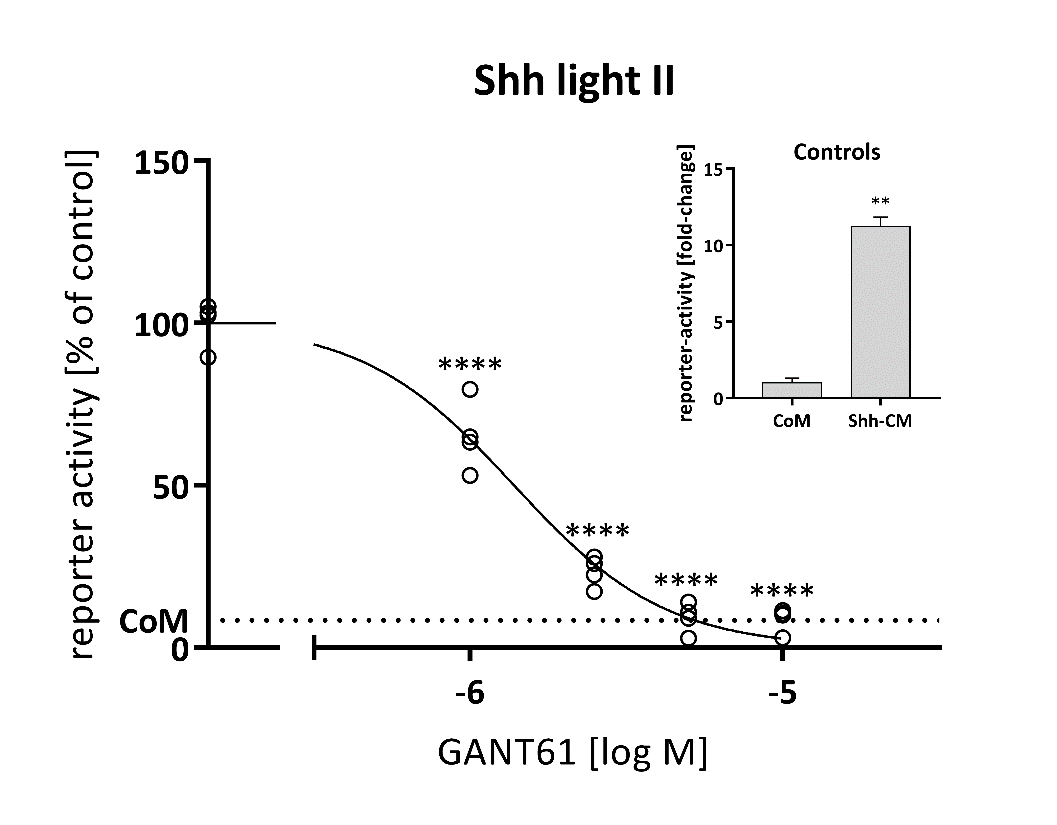


**Figure S3.** Functionality test of GANT61 using Shh light II. Shh light II were pre-incubated using Shh-CM for 24 h and treated with increasing doses of GANT (1 to 10 µM) for 48 h. Incubation of Shh-CM or CoM without treatment (Insert) was conducted accordingly. One experiment performed in 4 biological replicates. * *p* < 0.05; ** *p* < 0.01; *** *p* < 0.001; **** *p* < 0.0001 against solvent or control (insert).


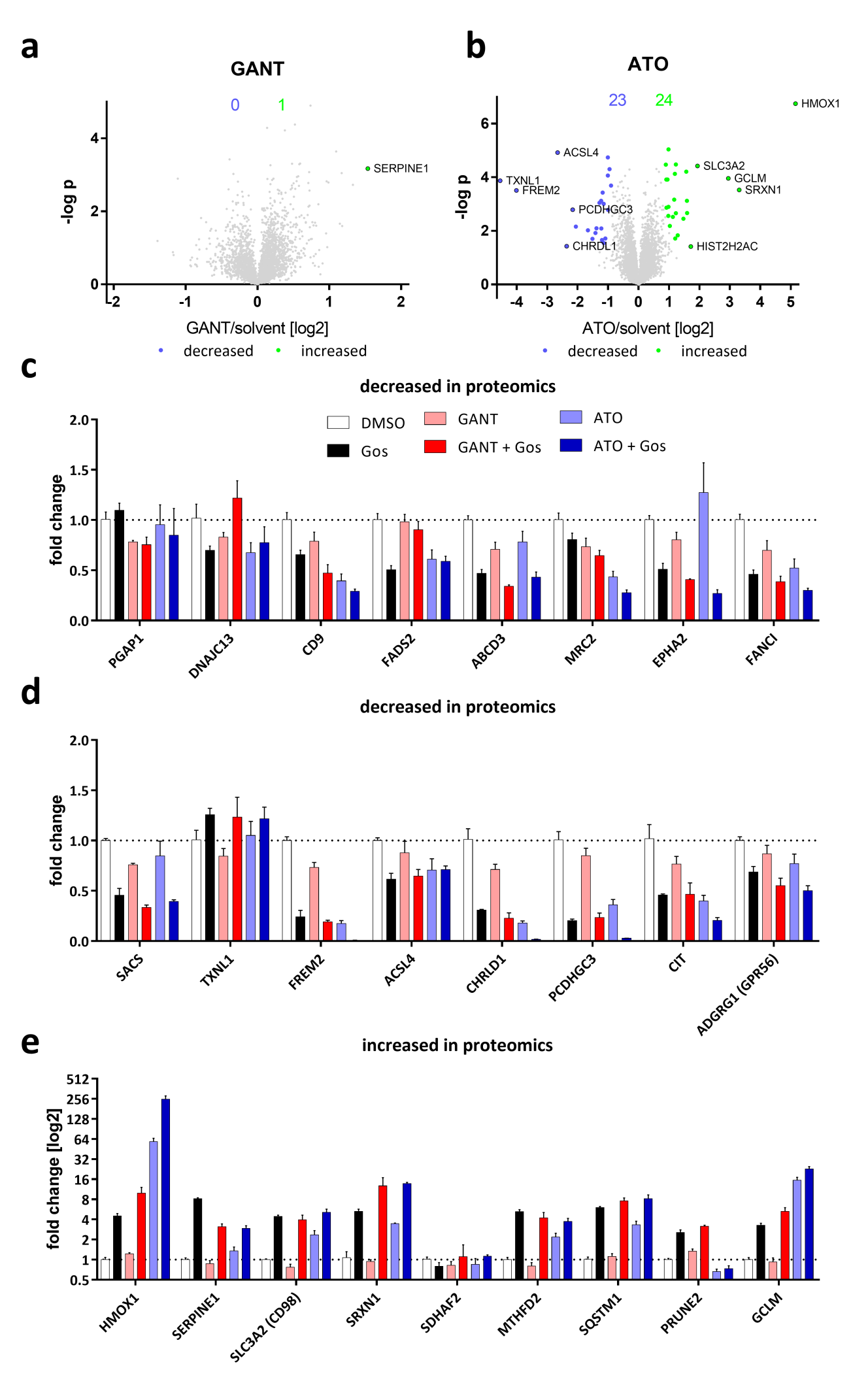


**Figure S4.** Proteomic changes after GANT61 and ATO treatment and Taqman-based validation of proteomic hits. (**a** and **b**) Volcano Plots showing the protein ratios (in log2) as function of the −log *p*-values of label-free quantification proteomics data of GS-5 after treatment for 24 h with (**a**) 2.5 µM GANT or (**b**) 2.5 µM ATO. The amount of significantly enriched protein is written above the plots (**c** to **e**). Bar graphs showing the relative quantification of the genes indicated after treatment with solvent (DMSO), Gos, GANT, ATO or a combination of GANT + Gos and ATO + Gos for 24 h. Gene expression values were normalized to TBP. Gene expression analysis was performed once in biological triplicates using Taqman-based probes.


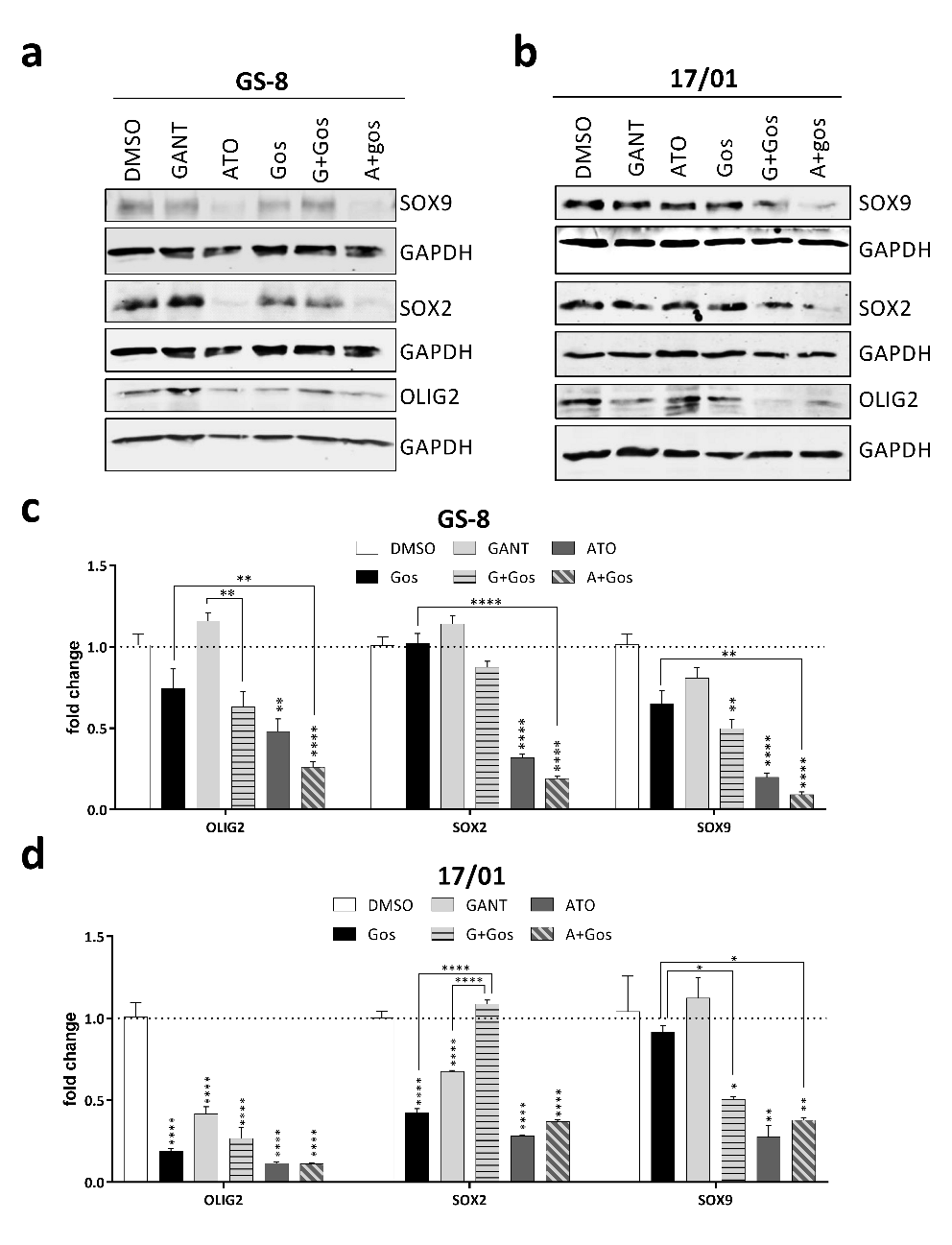


**Figure S5.** ATO, but not GANT in combination with Gos reduces GSC marker proteins and Hh and Notch pathway activity in GS-8 and 17/01. (**a** and **b**) Western Blot analysis of (**a**) GS-8 and (**b**) the primary line 17/01 after treatment for 24h with solvent (DMSO), 7.5 µM Gos, 5 µM GANT, 5 µM ATO or a combination of Gos with GANT (GANT + Gos) or ATO (ATO + Gos). (**c** and **d**) Taqman-based gene expression analysis of (**c**) GS-8 and (**d**) 17/01 after treatment for 24 h with 5 µM Gos, 2.5 µM GANT or ATO or the combination of GANT or ATO with Gos (GANT + Gos; ATO + Gos). The experiments were performed three times (GS-8) or once (17/01). The gene expression experiment were performed in 3 biological replicates. * *p* < 0.05; ** *p* < 0.01; *** *p* < 0.001; **** *p* < 0.0001; Two-way ANOVA followed by Tukey Post-Hoc-Test (GraphPad Prism 7).


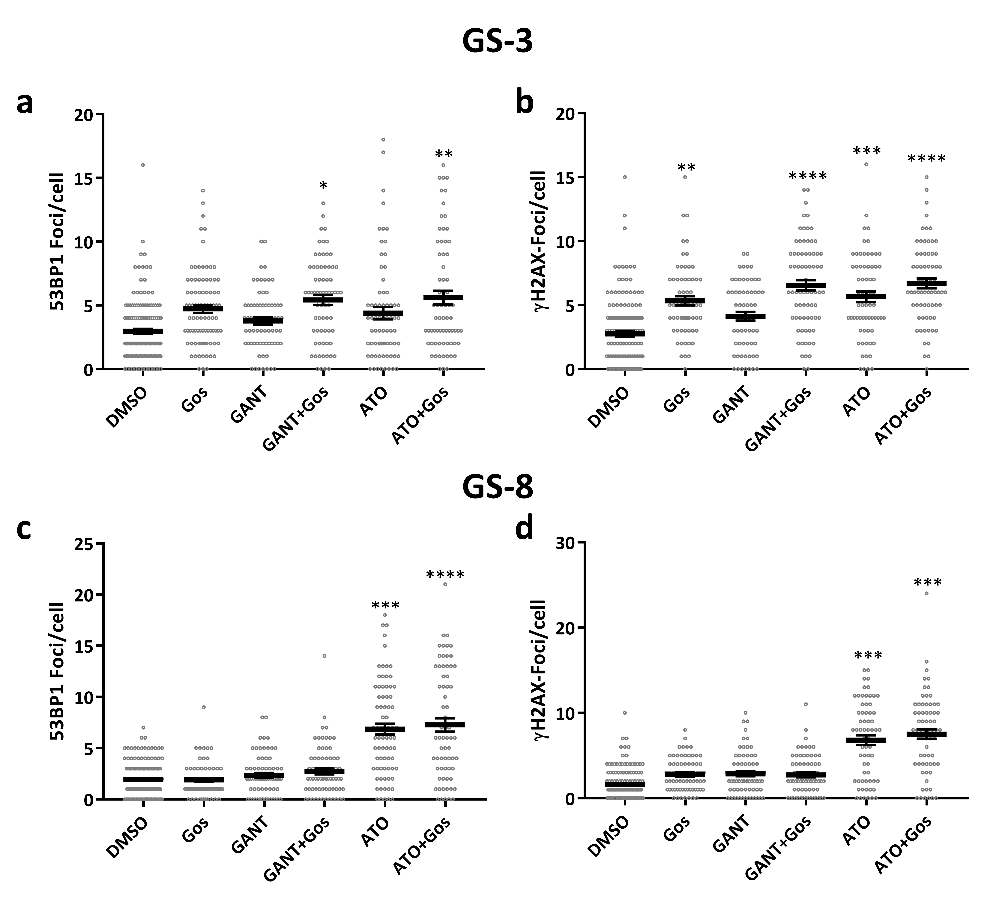


**Figure S6.** GANT or ATO in combination with Gos synergistically induce DNA damage in GSC. Dot-Plots of (**a** and **c**) TP53BP1- or (**b** and **d**) γH2AFX-positive foci per nucleus of (**a** and **b**) GS-3 or (**c** and **d**) GS-8 24h after treatment with 5 µM Gos, 3 µM GANT, 2.5 µM ATO or a combination of GANT and Gos (GANT + Gos) or ATO and Gos (ATO + Gos). Each point represents the number of foci per nucleus. The experiments were performed once in triplicates. For each replicate at least 20 nuclei were counted. Statistics were performed using the mean value of each replicate. * *p* < 0.05; ** *p* < 0.01; *** *p* < 0.001; **** *p* < 0.0001 against solvent; # *p* < 0.05 against both single treatments. One-way ANOVA followed by Tukey Post-Hoc-Test (GraphPad Prism 7).


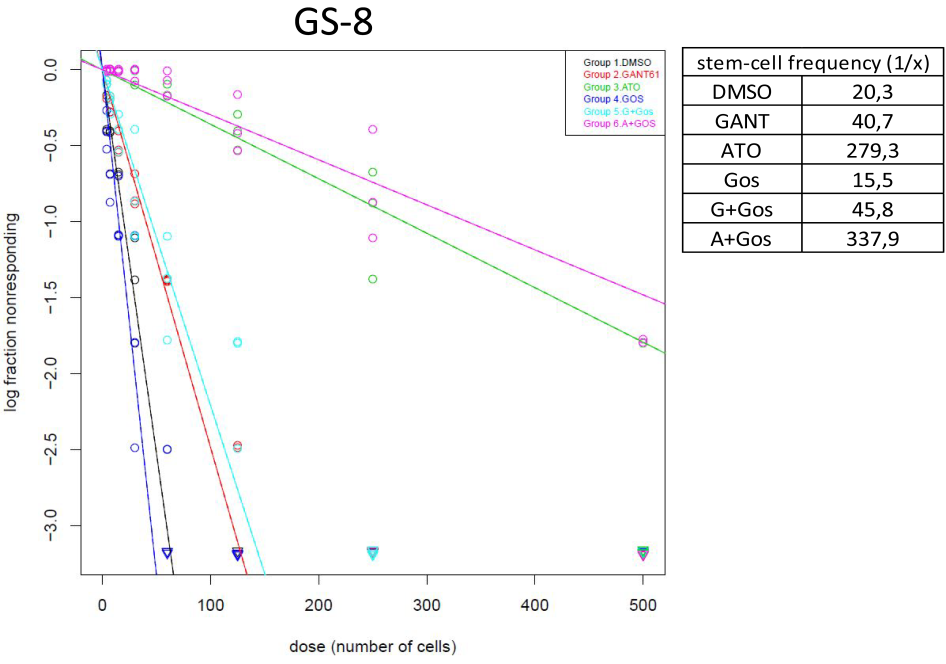


**Figure S7.** ATO treatment reduces stemness properties of GS-8 in vitro. (**a**) Extreme limiting dilution analysis [34] of GS-8 7 days after seeding and treatment with 1 µM ATO, 2.5 µM GANT61 or 0.5 µM Gos alone or in combinaton of increasing cell numbers (4 to 500 cells). The stem cell frequency calculated by ELDA software [34] is depicted below the graph.


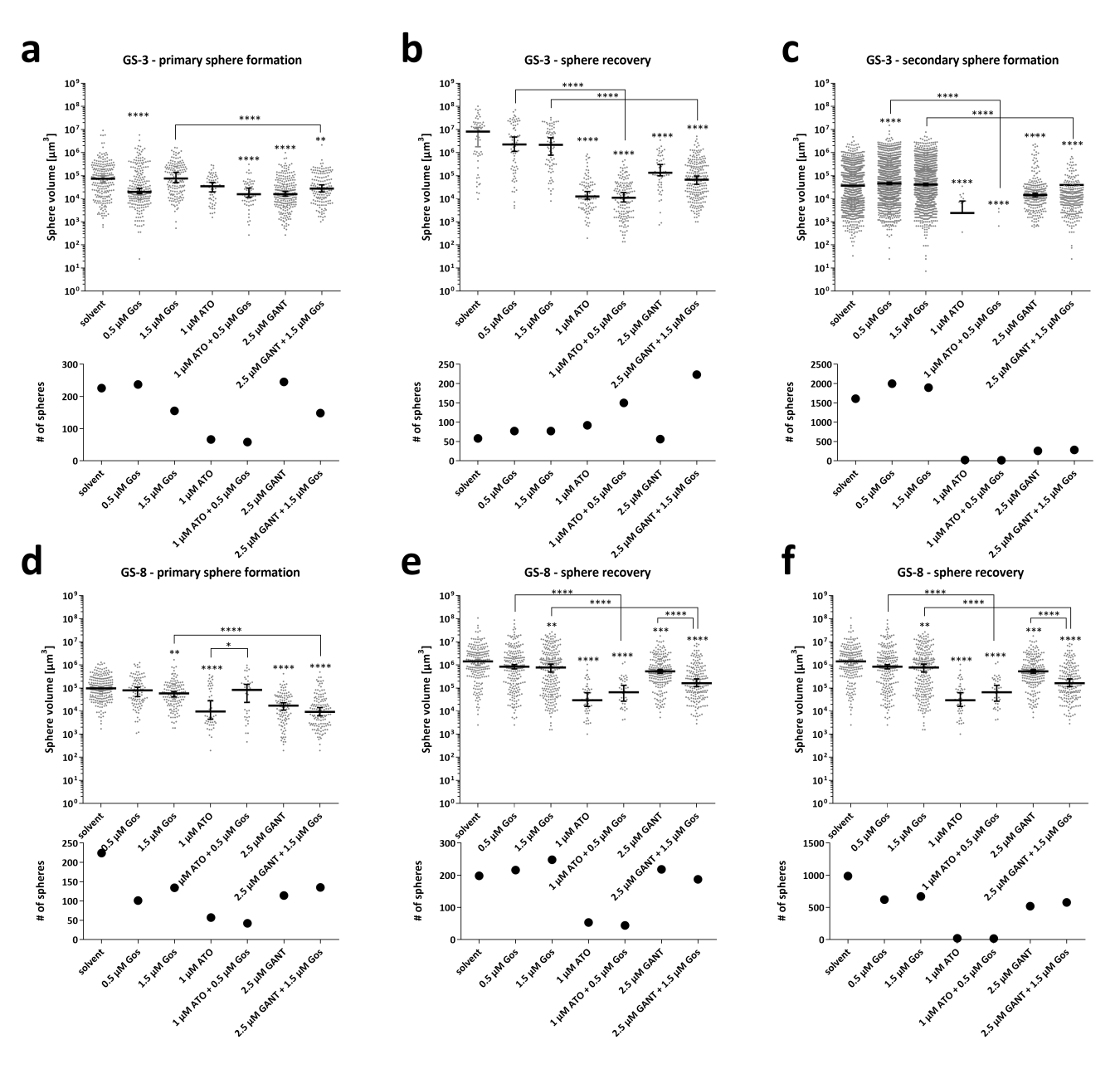


**Figure S8.** ATO treatment reduces stemness properties of GS-3 and GS-8 in vitro. Dot-Plots displaying the (upper row) sphere volume and (lower row) total number of spheres of primary spheres of (**a** to **c**) GS-3 and (**d** to **f**) GS-8 after measurement of (**a** and **d**) primary sphere formation, (**b** and **e**) sphere recovery and (**c** and **f**) secondary sphere formation. Horizontal lines are the median ± 95% confidence intervals. Limiting dilution assays were performed three times in 12 biological replicates. Sphere formation assays were performed twice in triplicates and three vision fields were analyzed for each biological replicate. * *p* < 0.05; ** *p* < 0.01; *** *p* < 0.001; **** *p* < 0.0001; One-way ANOVA followed by Tukey Post-Hoc-Test (GraphPad Prism 7).


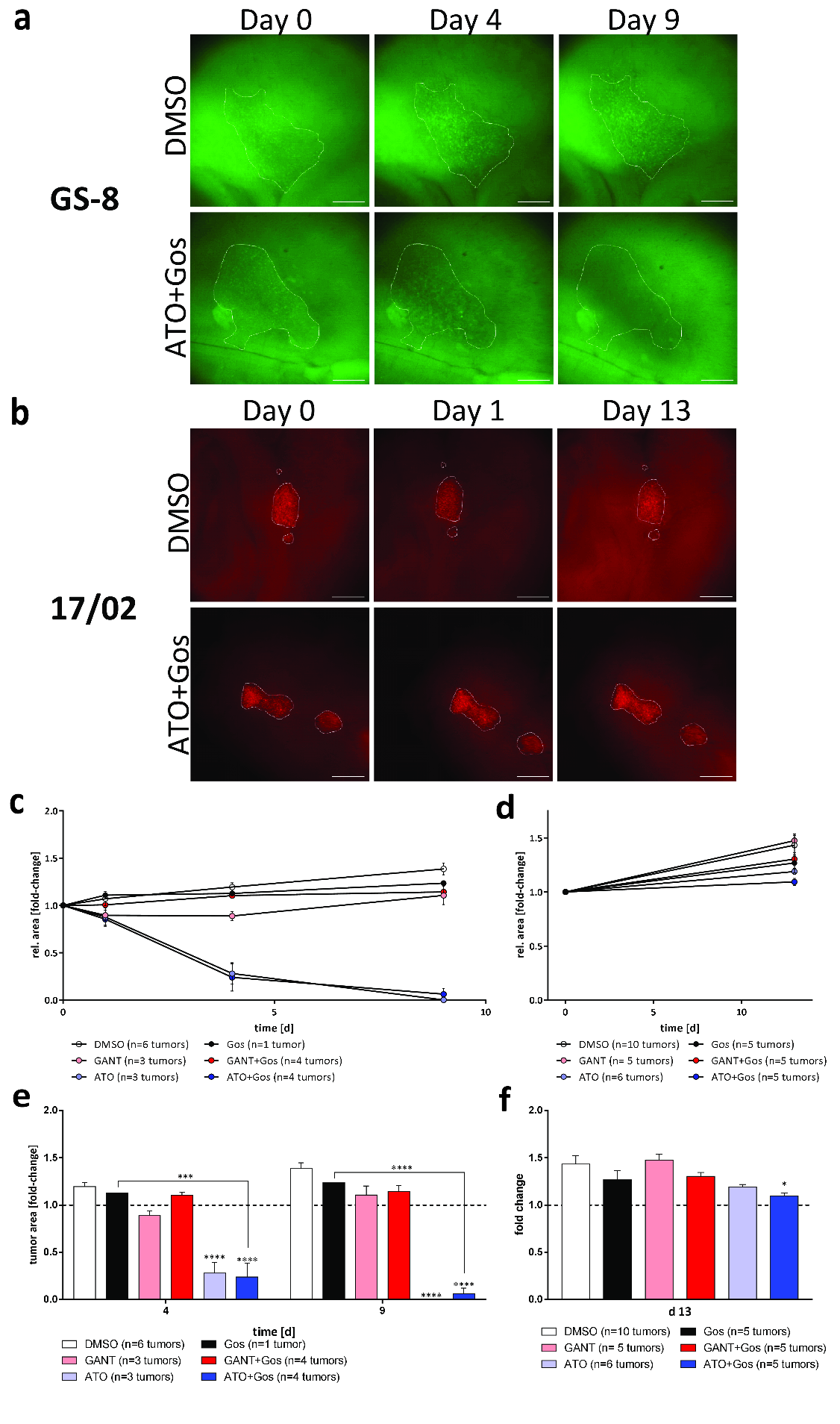


**Figure S9.** ATO and ATO/Gos decrease tumor size in adult OTC transplantation experiments of GS-8 GFP-Luc and 17/02. (**a** and **b**) Representative images of tumors of (**a**) GS-8 GFP-Luc and (**b**) CellTracker CM Dil-labeled primary cultures 17/02 grown on adult OTCs after treatment with solvent (DMSO) or ATO/Gos; scale bar: 500 µm. (**c** and **d**) Growth kinetic of (**c**) GS-8 GFP-Luc or (**d**) 17/02 tumors over time after treatment with solvent (DMSO, white), 2.5 µM GANT (pink), 2.5 µM ATO (light blue) or 5 µM Gos (black) or GANT/Gos (red) or ATO/Gos (blue). (E and F) Bar graph for selected time point displaying the mean (+ SEM) tumor size of (**e**) GS-8 GFP-Luc and (**f**) 17/02. * *p* < 0.05; ** *p* < 0.01; *** *p* < 0.001; **** *p* < 0.0001 compared to DMSO for each time-point. One-way ANOVA followed by Tukey Post-Hoc-Test (GraphPad Prism 7).

© 2019 by the authors. Licensee MDPI, Basel, Switzerland. This article is an open access article distributed under the terms and conditions of the Creative Commons Attribution (CC BY) license (http://creativecommons.org/licenses/by/4.0/).
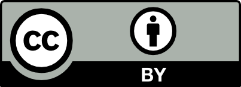

Supplement: Supplementary file 1 [file cancers-11-00350-s001.zip › cancers-426311-final-supplementary.docx]
